# Association of Malnutrition with Surgical and Hospital Outcomes after Spine Surgery for Spinal Metastases: A National Surgical Quality Improvement Program Study of 1613 Patients

**DOI:** 10.3390/jcm13061542

**Published:** 2024-03-07

**Authors:** Aladine A. Elsamadicy, John Havlik, Benjamin C. Reeves, Josiah J. Z. Sherman, Samuel Craft, Paul Serrato, Sumaiya Sayeed, Andrew B. Koo, Syed I. Khalid, Sheng-Fu Larry Lo, John H. Shin, Ehud Mendel, Daniel M. Sciubba

**Affiliations:** 1 Department of Neurosurgery, Yale University School of Medicine, 333 Cedar Street, New Haven, CT 06510, USA; 2 Department of Neurosurgery, University of Illinois at Chicago, Chicago, IL 60612, USA; 3 Department of Neurosurgery, Zucker School of Medicine at Hofstra, Long Island Jewish Medical Center, North Shore University Hospital, Northwell Health, Manhasset, NY 11030, USA; 4 Department of Neurosurgery, Massachusetts General Hospital, Harvard Medical School, Boston, MA 02114, USA

**Keywords:** malnutrition, spinal metastasis, NSQIP, length of stay, complication

## Abstract

**Background:** Malnutrition is a common condition that may exacerbate many medical and surgical pathologies. However, few have studied the impact of malnutrition on surgical outcomes for patients undergoing surgery for metastatic disease of the spine. This study aims to evaluate the impact of malnutrition on perioperative complications and healthcare resource utilization following surgical treatment of spinal metastases. **Methods:** We conducted a retrospective cohort study using the 2011–2019 American College of Surgeons National Surgical Quality Improvement Program database. Adult patients with spinal metastases who underwent laminectomy, corpectomy, or posterior fusion for extradural spinal metastases were identified using the CPT, ICD-9-CM, and ICD-10-CM codes. The study population was divided into two cohorts: Nourished (preoperative serum albumin values ≥ 3.5 g/dL) and Malnourished (preoperative serum albumin values < 3.5 g/dL). We assessed patient demographics, comorbidities, intraoperative variables, postoperative adverse events (AEs), hospital LOS, discharge disposition, readmission, and reoperation. Multivariate logistic regression analyses were performed to identify the factors associated with a prolonged length of stay (LOS), AEs, non-routine discharge (NRD), and unplanned readmission. **Results:** Of the 1613 patients identified, 26.0% were Malnourished. Compared to Nourished patients, Malnourished patients were significantly more likely to be African American and have a lower BMI, but the age and sex were similar between the cohorts. The baseline comorbidity burden was significantly higher in the Malnourished cohort compared to the Nourished cohort. Compared to Nourished patients, Malnourished patients experienced significantly higher rates of one or more AEs (Nourished: 19.8% vs. Malnourished: 27.6%, *p* = 0.004) and serious AEs (Nourished: 15.2% vs. Malnourished: 22.6%, *p* < 0.001). Upon multivariate regression analysis, malnutrition was found to be an independent and associated with an extended LOS [aRR: 3.49, CI (1.97, 5.02), *p* < 0.001], NRD [saturated aOR: 1.76, CI (1.34, 2.32), *p* < 0.001], and unplanned readmission [saturated aOR: 1.42, CI (1.04, 1.95), *p* = 0.028]. **Conclusions:** Our study suggests that malnutrition increases the risk of postoperative complication, prolonged hospitalizations, non-routine discharges, and unplanned hospital readmissions. Further studies are necessary to identify the protocols that pre- and postoperatively optimize malnourished patients undergoing spinal surgery for metastatic spinal disease.

## 1. Introduction

The rising healthcare expenditures and increased utilization of bundled payment programs in the United States have spurred interest in the development of more efficient patient care pathways that reduce costs while maintaining the quality of care [1,2]. To assess hospital performance regarding the quality of care and cost-saving measures, patients’ length of hospital stay (LOS) and odds of readmission have become indicators of the quality of patient care and efficiency of healthcare delivery [3,4]. Specifically, in spine surgery, a prolonged LOS and unplanned readmission following surgery have been found to be associated with a greater risk of complications [5], increased mortality [6], and greater healthcare expenditures [7]. One patient population that may benefit from value-based care initiatives is patients with metastatic disease of the spine who undergo spine surgery with an increasingly common endeavor of alleviating pain, restoring neurological function, or debulking the tumor from the spinal cord to allow for a more effective radiation therapy [8,9]. Approximately 10% of all patients with metastatic tumors will suffer from metastases in the spine, and the costs can be upward of USD 50,000 for the index hospitalization [10,11]. Thus, identifying patient-level factors associated with inferior outcomes following surgical treatment of spinal metastases would enable improved peri-operative risk stratification and patient optimization, improving patient care while decreasing unnecessary healthcare expenditures.

Malnutrition, a condition of muscle wasting due to a caloric deficit, is an increasingly common risk factor negatively influencing the outcomes of many medical and surgical pathologies [12]. It is particularly common in cancer patients, and some suggest that more than half of patients with spinal metastases present in a malnourished state [13]. Serum albumin has emerged as one of the most commonly used clinically relevant indicators of malnutrition due to its short half-life, with values of <3.5 g/dL frequently being defined as malnutrition in the literature [14,15]. While some studies have touched on the negative impact of malnutrition on outcomes among patients who undergo surgery for spinal metastases, the impact of patient malnutrition in this population remains largely understudied [16,17]. Moreover, as spinal metastases are often associated with disease progression and because of the high hospital costs associated with their interdisciplinary care, targeting ways to minimize both patient health exacerbation and hospital resource burden during the perioperative period are imperative [10,11].

The aim of this study was to evaluate the impact of malnutrition on perioperative complications and healthcare resource utilization following surgical treatment of spinal metastases. Specifically, we anticipate that this will have an effect on the length of stay, postoperative adverse events, non-routine discharge, and unplanned readmission; thus, we explored its relation to these outcomes.

## 2. Methods

### 2.1. Data Source

The American College of Surgeons (ACS) National Surgical Quality Improvement Program (NSQIP) database is a prospective, risk-adjusted database that examines 30-day postsurgical outcomes based on patient records.

### 2.2. Cohort Selection

NSQIP Participant Use Files from 2011 to 2019 were queried, and patients with spinal metastases using the International Classification of Disease, Ninth and Tenth Revision codes (ICD-9; 198.3-5, 733.13) and ICD-10 (C79.49, C79.51, M48.50X, M84.48X, and M84.68X) [1]. Using the Current Procedural Terminology (CPT) codes 22600, 22612, 22630, 22633, 63275-8, 63290, and 63300-3, patients undergoing laminectomy, corpectomy, or posterior arthrodesis for extradural spinal tumors were selected. All patients that were ≥18 years old were included. Those with preoperative transfusions, wound infections, and trauma were excluded. Surgeries without the intention of tumor removal, such as fixation alone, were not included. Any cases not operated on by neurosurgeons or orthopedic surgeons, or not performed under general anesthesia were excluded. The study population was divided into two cohorts: Nourished (preoperative serum albumin values ≥ 3.5 g/dL) and Malnourished (preoperative serum albumin values < 3.5 g/dL).

### 2.3. Patient Characteristics and Outcomes

The demographic information assessed included age, sex, race, and body mass index (BMI). Patient comorbidities included ASA (American Society of Anesthesiologists) grade, electrolyte abnormalities (preoperative Na < 135 mEq/L or >145 mEq/L), renal failure, emergency case status, smoking, diabetes mellitus (DM), dyspnea, chronic lung disease, congestive heart failure (CHF), dependent functional status, hypertension (HTN), chronic steroid use, bleeding disorders, and anemia. Perioperative variables of interest included the procedure type (corpectomy, fusion, or laminectomy), total operative time, and red blood cell (RBC) transfusion on the day of surgery.

Postoperative AEs within the first 30 postoperative days were grouped as surgical (superficial surgical site infection (SSI), deep SSI, organ space SSI, and wound dehiscence) and medical (pneumonia (PNA), unplanned reintubation, ventilator requirements, pulmonary embolism (PE), renal insufficiency, acute renal failure (ARF), urinary tract infection (UTI), cardiac arrest/MI, deep vein thrombosis (DVT), *Clostridium difficile* colitis (*C. diff* colitis), systemic sepsis, or septic shock) adverse events. AEs were also categorized according to severity as minor or severe AEs (MAE and SAE, respectively). Superficial SSI, UTI, PNA, and renal insufficiency were categorized as MAE, while SAE consisted of all other AEs and returns to the operating room. The healthcare resource utilization outcomes analyzed in this study were the LOS, non-routine discharge (NRD), reoperation, and readmission. Readmission was stratified into unplanned readmission (vs. planned readmission), and unplanned readmission was further classified as related to the principal procedure (vs. unrelated to the principal procedure).

### 2.4. Statistical Analysis

Data segmentation and statistical analysis were performed in Stata, version 16.1 (StataCorp, College Station, TX, USA). Categorical variables were expressed as rate percentages and compared using chi-squared tests if all cells had counts >5 or the Fisher exact tests if any cells had counts ≤5, while continuous variables were expressed as the means with standard deviations (SDs) or medians with interquartile ranges (IQRs), and compared using the Student *t*-tests if normally distributed or the nonparametric Wilcoxon rank-sum (Mann–Whitney) *U* test if nonnormally distributed. Shapiro–Wilk tests were employed to test for normality of continuous variables.

### 2.5. Univariate and Multivariate Analyses

Variables that significantly differed between the two cohorts in previous statistical analysis and had N > 9 were included in univariate and multivariate regressions to model predictors of the total hospital LOS, NRD, the occurrence of any AE, and unplanned readmissions. Linear regression models were constructed for the total LOS to calculate the crude and adjusted relative risks (RRs and aRRs) with 95% confidence intervals (CIs). Logistic regression models were constructed for NRD, the presence of any AEs, and unplanned readmissions to calculate the crude and adjusted odds ratios (ORs and aORs) with CIs. All tests were two-sided, and significance was set at *p* < 0.05.

## 3. Results

### 3.1. Patient Demographics

A total of 1613 patients were identified, of whom 1193 (74.0%) were Nourished and 420 patients (26.0%) were Malnourished (Table 1). The mean patient age was similar between the Nourished and Malnourished cohorts (Nourished: 61.8 ± 13.0 years vs. Malnourished: 63.1 ± 12.0 years, *p* = 0.124), as was the percentage of female patients (Nourished: 42.8% vs. Malnourished: 37.9%, *p* = 0.075) (Table 1). Patient race varied significantly between the cohorts, with a greater proportion of Malnourished patients being African American compared to the Nourished cohort (Nourished: 11.7% vs. Malnourished: 15.0%, *p* = 0.006) (Table 1). The Malnourished cohort had a significantly lower mean BMI than the Nourished cohort (Nourished: 27.6 ± 6.3 kg/m^2^ vs. Malnourished: 26.6 ± 6.5 kg/m^2^, *p* = 0.004) (Table 1).

### 3.2. Prevalence of Patient Comorbidities

Malnourished patients had significantly greater ASA classification scores compared to Nourished patients, with a greater proportion of patients in the Malnourished cohort having a score of three or more (Nourished: 84% vs. Malnourished: 93.3%, *p* < 0.001) (Table 1). Compared to Nourished patients, a significantly greater proportion of Malnourished patients had comorbid electrolyte abnormalities (Nourished: 15.8% vs. Malnourished: 25.0%, *p* < 0.001), emergency case status (Nourished: 17.0% vs. Malnourished: 21.4%, *p* = 0.044), smoking (Nourished: 18.9% vs. Malnourished: 24.5%, *p* = 0.015), dyspnea (Nourished: 5.5% vs. Malnourished: 11.2%, *p* < 0.001), CHF (Nourished: 0.2% vs. Malnourished: 1.4%, *p* = 0.005), dependent functional status (Nourished: 9.3% vs. Malnourished: 13.4%, *p* = 0.016), bleeding disorders (Nourished: 4.7% vs. Malnourished: 10.0%, *p* < 0.001), and anemia (Nourished: 55.3% vs. Malnourished: 79.5%, *p* < 0.001) (Table 1). The rates of the other comorbidities were similar between the cohorts, including renal failure (*p* > 0.99), diabetes mellitus (*p* = 0.853), chronic lung disease (*p* = 0.273), hypertension (*p* = 0.710), and steroid use (*p* = 0.055) (Table 1).

### 3.3. Intraoperative Variables

Malnourished and Nourished patients underwent specific procedures at similar rates (*p* = 0.436), including corpectomy (Nourished: 7.2% vs. Malnourished: 6.4%), fusion (Nourished: 25.5% vs. Malnourished: 22.9%) and laminectomy (Nourished: 67.3% vs. Malnourished: 70.7%) (Table 2). The total operation time was similar between the cohorts (Nourished: 206.0 ± 108.3 min vs. Malnourished: 213.4 ± 129.1 min, *p* = 0.903), though the rate of red blood cell transfusion on the day of surgery was significantly greater in the Malnourished cohort compared to the Nourished cohort (Nourished: 22.7% vs. Malnourished: 33.6%, *p* < 0.001) (Table 2).

### 3.4. Thirty-Day Adverse Events (AEs), Reoperations, and Readmissions

Regarding surgical and medical AEs, a significantly greater proportion of Malnourished patients experienced wound dehiscence (Nourished: 0.4% vs. Malnourished: 1.4%, *p* = 0.031), deep vein thrombosis (Nourished: 3.9% vs. Malnourished: 6.4%, *p* = 0.029), and systemic sepsis (Nourished: 1.4% vs. Malnourished: 3.3%, *p* = 0.014) compared to Nourished patients (Table 2). Compared to Nourished patients, a significantly greater proportion of Malnourished patients experienced one or more AEs (Nourished: 19.8% vs. Malnourished: 27.6%, *p* = 0.004) and AEs classified as serious (Nourished: 15.2% vs. Malnourished: 22.6%, *p* < 0.001) (Table 2). Malnourished patients experienced longer hospital stays (Nourished: 6.7 ± 15.9 days vs. Malnourished: 11.8 ± 14.4 days, *p* < 0.001), greater rates of NRD (Nourished: 39.1% vs. Malnourished: 55.9%, *p* < 0.001), and unplanned readmission (Nourished: 12.6% vs. Malnourished: 20.5%, *p* < 0.001) compared to the Nourished cohort, though reoperation rates were similar between the cohorts (Nourished: 6.0% vs. Malnourished: 6.9%, *p* = 0.527) (Table 2).

### 3.5. Multivariate Analyses

Malnourishment was an independent predictor of an extended LOS on multivariate regression analysis [aRR: 3.49, CI (1.97, 5.02), *p* < 0.001] (Table 3). RBC transfusion on the day of surgery was a significant predictor of an extended LOS (*p* = 0.008) (Table 3).

While malnourishment was associated with an increased complication rate upon univariate analysis, this difference was not statistically significant for a multivariate analysis [aOR: 1.26, CI (0.96, 1.65), *p* = 0.101] (Table 4). On multivariate regression analysis, independent predictors of increased AEs included female sex (*p* = 0.018), ASA 3–4 (*p* = 0.010), emergency case status (*p* = 0.001), baseline dyspnea (*p* = 0.009), and preoperative anemia (*p* = 0.005) (Table 4).

On multivariate regression analysis, malnourishment was an independent predictor of NRD [saturated aOR: 1.76, CI (1.34, 2.32), *p* < 0.001] (Table 5). Other significant predictors of NRD included increased age (*p* < 0.001), African American race (*p* = 0.002), emergency case status (*p* < 0.001), dependent functional status (*p* < 0.001), bleeding disorder (*p* = 0.002), and RBC transfusion on the day of surgery (*p* = 0.010) (Table 5).

On multivariate regression analysis, malnourishment was an independent predictor of unplanned readmission [saturated aOR: 1.42, CI (1.04, 1.95), *p* = 0.028] (Table 6). Increased age (*p* = 0.033), bleeding disorder (*p* = 0.012), anemia (*p* = 0.001), DVT (*p* < 0.001), and systemic sepsis (*p* = 0.024) were also independent predictors of unplanned readmission (Table 6).

## 4. Discussion

In this retrospective cohort study of 1613 patients who underwent spine surgery for metastatic disease using the ACS-NSQIP database, we found that both the prevalence and severity of postoperative AEs, LOS, NRD rates, and unplanned readmission rates were greater in Malnourished patients compared to nourished patients. Additionally, upon multivariate analysis, we found that malnourishment was an independent predictor of an increased LOS, NRD, and unplanned hospital readmission.

A number of studies have sought to identify the prevalence of malnutrition in patients undergoing surgical treatment for spinal metastases. One retrospective study of 4583 adult patients undergoing surgery for spinal metastases secondary to breast, lung, thyroid, renal, or prostate cancer by De La Garza Ramos et al. found that 3.8% of patients were malnourished [18]. In another retrospective study of 479 patients undergoing surgery for metastatic spinal cancer at a single institution, Massaad et al. demonstrated that 10.6% of patients were malnourished [19]. However, other studies have observed substantially higher rates of malnutrition in the spinal oncology population. In Hussain et al.’s retrospective cohort study of 1498 adult patients undergoing laminectomy and excision of metastatic extradural tumors from 2011 to 2014, the authors found that 34.2% of patients had preoperative hypoalbuminemia [20]. Similarly, in a retrospective cohort study of 95 patients undergoing surgical treatment of spinal metastasis from 2009 to 2011 at a university teaching hospital, Kumar et al. found that 42.1% of patients had pathologically low serum albumin levels prior to surgery [21]. Likewise, in a retrospective cohort study of 95 patients who underwent spine surgery for metastases at a tertiary care center from 2008 to 2016, Ehresman et al. demonstrated that 52.6% of patients were moderately-to-severely malnourished [13]. In our study, we found that 26.0% of patients undergoing surgery for spinal metastases were malnourished. This variability in the reported rates of preoperative malnutrition highlights the need for additional studies to characterize the prevalence of malnutrition more accurately.

The high prevalence of malnutrition in patients undergoing surgery for spinal metastases is particularly concerning given the negative impact malnutrition may have on postoperative complications. In the study of 1498 patients who underwent laminectomy for metastatic extradural spinal tumors, Hussain et al. found that malnutrition was an independent risk factor for sepsis and intra-operative or postoperative transfusion, and that moderate-to-severe or severe hypoalbuminemia was an independent risk factor for any complication [20]. Likewise, in a retrospective cohort study of 1176 patients who underwent spine surgery for metastatic disease of the spine, Boaro et al. demonstrated that hypoalbuminemia was significantly associated with both major and minor postoperative complications [22]. Similarly, De La Garza Ramos et al. demonstrated that malnutrition was an independent predictor of incidence of at least one postoperative complication, and malnutrition was subsequently chosen as a key variable in their novel risk stratification model designed to predict complications following surgery for spinal metastases [18]. Using logistic regression, random forest, and gradient-boosted decision tree models, Massaad et al. found that malnutrition was one of the strongest predictors of complications in a study of 479 patients undergoing surgery for metastatic spinal tumor [19]. Likewise, in a study of 95 patients who underwent spine surgery for metastases, Ehresman et al. found that moderate-to-severe malnourishment was an independent predictor of 30-day complications [13]. Similar to the aforementioned studies, we found that malnutrition was associated with both the number of complications encountered following surgery, as well as the severity of postoperative complications. Therefore, it seems warranted that improved peri-operative nutrition optimization in malnourished patients may play a role in reducing postoperative complications and may improve quality of patient care while decreasing unnecessary healthcare expenditures.

Similar to its negative impact on postoperative complications, baseline malnutrition has been shown to lead to longer hospital stays for patients undergoing surgery for spinal metastases. In the study by Hussain et al. of patients undergoing laminectomy for metastatic extradural spinal tumors, the authors found that malnutrition was an independent risk factor for a prolonged LOS [20]. Similarly, in a study of 95 patients who underwent spine surgery for metastases, Ehresman et al. showed that moderate-to-severe malnourishment was an independent predictor of extended hospital stay [13]. Likewise, in a retrospective cohort study of 350 patients undergoing surgery for primary or secondary vertebral column tumors over a 46 month period, Ehresman et al. demonstrated that patients with an LOS > 10 days had significantly lower serum albumin levels (*p *< 0.001) than those with an LOS < 10 days, and that lower serum albumin levels was associated with more days at the hospital upon univariate analysis (*p *< 0.001) [16]. Similar to the aforementioned studies, we found that malnutrition was an independent predictor of an increased LOS upon multivariate regression analysis.

Previous studies have sought to determine the impact of baseline malnutrition on other hospital quality of care metrics such as NRD, unplanned readmission, and mortality. In a study of 1498 patients who underwent laminectomy for metastatic extradural spinal tumors, Hussain et al. found that hypoalbuminemia was an independent risk factor for non-home discharge disposition and 30-day mortality, had no impact on reoperation rates, and, interestingly, was protective against readmission [20]. In contrast, in a retrospective NSQIP study of 2207 patients undergoing surgery for primary and secondary spinal tumors from 2011 to 2014, Karhade et al. found that serum albumin <3.5 g/dL was a significant risk factor for 30-day readmission upon univariate analysis (OR:1.81, *p* = 0.001) [17]. Likewise, in a study of 350 patients undergoing surgery for vertebral column tumors, Ehresman et al. observed that patients discharged home had significantly higher serum albumin levels than those discharged to non-home destinations (*p* < 0.001), and that lower serum albumin levels were an independent predictor of NRD (*p* < 0.001) [16]. In a retrospective cohort study of 88 patients who were surgically treated for renal cell carcinoma metastases of the spinal column, Massaad et al. found that a lower nutritional index was associated with worse overall survival (*p* = 0.003) [23]. In a retrospective cohort study of 700 patients who underwent surgery for metastatic spine disease from 2006 to 2016, Gelfand et al. found that patients with lower serum albumin levels experienced significantly higher rates of mortality compared to patients with higher serum albumin levels, and that serum albumin <2.5 g/dL was an independent predictor of 30-day mortality (OR: 6.2, *p* < 0.001) [24]. In the present study, we found that preoperative malnutrition was an independent predictor of NRD and unplanned readmission on multivariate regression analysis. While malnutrition should not preclude patients from undergoing surgical management of metastatic tumors, preoperative risk-stratification and optimization protocols should be implemented to improve patient outcomes in this high-risk population.

Given the negative impact of malnutrition on postoperative outcomes in patients undergoing surgery for spinal metastases, some have sought to identify malnutrition preoperatively with varying results. Besides hypoalbuminemia, the Global Leadership Initiative on Malnutrition (GLIM) criteria, which include the assessment of body mass, food intake, and disease burden, have been utilized to identify malnutrition and have been associated with mortality and complications [25]. Another metric, the geriatric nutrition risk index combined with calf circumferences (GNRI-CC), which measures many variables including albumin and calf length, has also been shown to be a predictor of prognosis in oncology patients undergoing surgery [26]. In a study of 95 patients who underwent spine surgery for metastases, Ehresman et al. found that patients who received a preoperative nutrition consult from a trained dietitian within twelve weeks of surgery experienced reduced hospital stays and 30-day complication rates [13]. In contrast, in a retrospective study of 68 patients undergoing posterior instrumented fusion for neuromuscular spinal deformity (a patient population of which the prevalent malnourishment and low BMI are cited as reasons for poor postoperative outcomes), Meltzer-Bruhn et al. found that preoperative nutrition consultation up to one year prior to surgery did not lead to weight optimization [27]. Despite these mixed results, assessing malnutrition preoperatively, potentially including preoperative nutritionist consults, may help reduce adverse outcomes. Given the many modalities of assessing malnutrition, a combination of nutritional consults, measurement of body mass and calf circumferences, serum albumin, and quantified food intake could help stratify patients into different risk level groups. These, in conjunction with the disease burden, could serve as indicators for how poorly these patients will tolerate surgery and how aggressively their nutritional status should be addressed before undergoing operative treatment.

After assessing nutritional status in patients, addressing the needs of malnourished patients is crucial. In a randomized control trial by Saleh et al. of 103 malnourished patients undergoing spine surgery, those who received perioperative nutritional supplementation in the form of protein shakes experienced lower rates of minor and wound-related complications, while those who did not had higher rates of requiring repeat surgery [28]. In a review by Williams et al., the recommended steps for nutritional optimization included high protein and carbohydrate intake and immunonutrition, or incorporating high amounts of various amino acids and fish oil, as studies have demonstrated their beneficial effects to patients [29]. Thus, optimizing nutritional status can include meeting caloric requirements, focusing on protein sources, and a discussion with patients and families on barriers to achieving these goals. These varied results highlight the need for additional studies to develop and refine preoperative patient optimization protocols and bolster preoperative diet to improve the quality of patient care and lower healthcare utilization.

A few aspects of this database study may limit this study’s interpretation and generalizability. First, the retrospective nature of analysis limits the ability to make conclusions about causation. Second, with data available only by ICD coding diagnosis and CPT procedural codes, there is a possibility of misclassified or incomplete data, coding and reporting biases, and exclusion of non-coded information, such as nutritional interventions. Next, while hypoalbuminemia has been validated and used many times in the literature as a metric for malnutrition, patient circumstances in real life can be much more complex, and ideally, a multifactorial means of deeming a patient malnourished would further improve the characterization of malnutrition. Furthermore, different staging procedures during a hospitalization may be co-coded, possibly leading to duplications. Finally, while a benefit of the NSQIP database is that complications are recorded after hospital discharge, only those that occur within the first 30 days are included in the database. Despite these limitations, this study elucidates the relationship between preoperative malnutrition and outcomes following surgical treatment of spinal metastases.

## 5. Conclusions

Our study suggests that patients who were malnourished tended to have more comorbidities, more AEs, and longer hospitalizations. Furthermore, malnutrition increases the risk of postoperative complication, prolonged hospitalizations, non-routine discharges, and unplanned hospital readmissions, even when other patient or procedural factors are accounted for, as our multivariate analyses indicate. Further studies are necessary to identify malnutrition and implement protocols that pre- and postoperatively optimize malnourished patients undergoing spinal surgery for metastatic spinal disease.

## Figures and Tables

**Table 1:** Patient demographics and comorbidities.

Variable	Nourished (n = 1193)	Malnourished (n = 420)	*p*-Value
**Age (Years)**			0.124
Mean ± SD	61.8 ± 13.0	63.1 ± 12.0	
Median [IQR]	63.0 [55.0–71.0]	64.0 [56.5–71.0]	
**Female (%)**	42.8	37.9	0.075
**Race (%)**			0.006 *
NHW	77.0	68.1	
NHB	11.7	15.0	
Hispanic	6.3	10.6	
Asian	4.3	6.4	
AIAN	0.3	0.0	
NHOPI	0.4	0.0	
**BMI**			0.004 *
Mean ± SD	27.6 ± 6.3	26.6 ± 6.5	
Median [IQR]	26.9 [23.6–31.2]	25.9 [22.4–30.0]	
**ASA classification**			<0.001 *
1	0.4	0.2	
2	15.5	6.4	
3	66.4	67.1	
4	17.6	26.2	
Electrolyte abnormality	15.8	25.0	<0.001 *
Renal failure	0.3	0.2	>0.99
Emergency case	17.0	21.4	0.044 *
Smoking	18.9	24.5	0.015 *
DM	12.5	12.1	0.853
Dyspnea	5.5	11.2	<0.001 *
Chronic lung disease	5.5	6.9	0.273
CHF	0.2	1.4	0.005 *
Dependent functional status	9.3	13.4	0.016 *
HTN	48.2	47.1	0.710
Steroids	15.3	19.3	0.055
Bleeding disorder	4.7	10.0	<0.001 *
Anemic	55.3	79.5	<0.001 *

SD: standard deviation; IQR: interquartile range; NHW: non-Hispanic white; NHB: non-Hispanic black; AIAN: American Indian or Alaska native; NHOPI: native Hawaiian or Pacific islander; BMI: body mass index; ASA: American Society of Anesthesiologists; DM: diabetes mellitus; CHF: congestive heart failure; HTN: hypertension. * statistically significant.

**Table 2:** Intraoperative variables, 30-day adverse events (AEs), reoperations, and readmissions.

Variable	Nourished (n = 1193)	Malnourished (n = 420)	*p*-Value
**Procedure type (%)**			0.436
Corpectomy	7.2	6.4	
Fusion	25.5	22.9	
Laminectomy	67.3	70.7	
**Total operation time (min)**			0.903
Mean ± SD	206.0 ± 108.3	213.4 ± 129.1	
Median [IQR]	182.0 [131.0–255.0]	180.5 [127.0–269.5]	
**RBC transfusion day of surgery (%)**	22.7	33.6	<0.001 *
**Surgical AEs (%)**			
Superficial SSI	1.5	1.9	0.579
Deep incisional SSI	1.1	0.7	0.775
Organ space SSI	0.3	1.0	0.080
Wound dehiscence	0.4	1.4	0.031 *
**Medical AEs (%)**			
PNA	3.5	5.7	0.051
Reintubation	1.5	1.9	0.579
Ventilator requirement	1.5	1.4	0.907
PE	2.0	3.1	0.202
Renal insufficiency	0.3	0.5	0.654
ARF	0.2	0.0	>0.99
UTI	2.6	2.6	0.982
Cardiac arrest or MI	1.1	1.0	>0.99
DVT	3.9	6.4	0.029 *
C. diff colitis	0.6	1.1	0.433
Systemic sepsis	1.4	3.3	0.014 *
Septic shock	0.8	1.0	0.766
Postoperative RBC transfusion (%)	5.5	6.2	0.571
**Number of AEs (%)**			0.004 *
0 AE	80.2	72.4	
1 AE	14.1	20.0	
>1 AE	5.7	7.6	
**AE severity (%)**			
MAE	7.5	10.2	0.098
SAE	15.2	22.6	<0.001 *
**LOS (days)**			<0.001 *
Mean ± SD	6.7 ± 15.9	11.8 ± 14.4	
Median [IQR]	7.0 [4.0–10.0]	10.0 [7.0–16.0]	
**Non-routine discharge (%)**	39.1	55.9	<0.001 *
**Reoperation (%)**	6.0	6.9	0.527
**Readmission (%)**			
Unplanned readmission	12.6	20.5	<0.001 *
Relevance to the principal procedure	8.3	11.7	0.040 *

RBC: red blood cell; AE: adverse event; SSI: surgical site infection; PNA: pneumonia; PE: pulmonary embolism; ARF: acute renal failure; UTI: urinary tract infection; MI: myocardial infarction; DVT: deep vein thrombosis; C. diff: Clostridium difficile; MAE: minor adverse event; SAE: serious adverse event; LOS: length of stay; SD: standard deviation; IQR: interquartile range. * statistically significant.

**Table 3:** Impact of preoperative variables on the length of stay. Preoperative variables were included in univariate analysis if significant in Table 1, Table 2 and Table 3 and N > 9. Variables were included in the multivariate model if *p* < 0.2 for univariate analysis.

Variable	Univariate		Multivariate	
	RR (CI)	*p*-Value	aRR (CI)	*p*-Value
Malnourished	5.09 (3.36–6.81)	<0.001 *	3.49 (1.97–5.02)	<0.001 *
Age	−0.03 (−0.09–0.03)	0.264	−0.01 (−0.06–0.04)	0.740
Female	−0.10 (−1.65–1.46)	0.904	−0.16 (−1.55–1.23)	0.820
**Race**				
NHW	−1.47 (−2.97–0.03)	0.055	REF	REF
NHB	1.61 (−0.37–3.58)	0.110	1.39 (−0.61–3.39)	0.173
Hispanic	0.66 (−1.83–3.15)	0.604	0.34 (−2.17–2.85)	0.793
Asian	1.63 (−1.42–4.68)	0.295	1.54 (−1.56–4.64)	0.330
AIAN	−5.07 (−18.92–8.79)	0.473	−3.93 (−17.67–9.80)	0.574
NHOPI	−2.40 (−14.40–9.61)	0.695	−0.70 (−12.61–11.21)	0.908
BMI	−0.05 (−0.17–0.07)	0.413	-	-
ASA (1–2)	−2.57 (−4.80–−0.35)	0.024 *	REF	REF
ASA (3–4)	2.57 (0.35–4.80)	0.024 *	1.56 (−0.46–3.58)	0.130
Electrolyte abnormality	1.50 (−0.49–3.48)	0.139	0.03 (−1.69–1.75)	0.971
Emergency case	−0.83 (−2.82–1.15)	0.411	-	-
Smoking	−0.86 (−2.76–1.04)	0.374	-	-
Dyspnea	0.69 (−2.31–3.69)	0.652	-	-
Dependent functional status	−0.51 (−3.04–2.01)	0.690	-	-
Bleeding disorder	2.59 (−0.61–5.80)	0.112	1.79 (−0.91–4.49)	0.194
Anemic	2.22 (0.63–3.80)	0.006 *	1.10 (−0.36–2.55)	0.139
RBC transfusion day of surgery	3.01 (1.26–4.76)	0.001 *	2.06 (0.54–3.59)	0.008 *

RR: relative risk; CI: 95% confidence interval; aRR: adjusted relative risk; NHW: non-Hispanic white; NHB: non-Hispanic black; AIAN: American Indian or Alaska native; NHOPI: native Hawaiian or Pacific islander; BMI: body mass index; ASA: American Society of Anesthesiologists classification; RBC: red blood cell. * statistically significant.

**Table 4:** Impact of preoperative variables on the odds of any complication. Preoperative variables were included in univariate analysis if significant in Table 1, Table 2 and Table 3 and N > 9. Variables were included in the multivariate model if *p* < 0.2 for univariate analysis.

Variable	Univariate		Multivariate	
	OR (CI)	*p*-Value	aOR (CI)	*p*-Value
Malnourished	1.55 (1.20–2.00)	0.001 *	1.26 (0.96–1.65)	0.101
Age	1.01 (1.00–1.02)	0.047 *	1.01 (1.00–1.02)	0.108
Female	1.17 (0.93–1.49)	0.187	1.37 (1.06–1.77)	0.018 *
**Race**				
NHW	0.95 (0.71–1.28)	0.745	-	-
NHB	1.25 (0.86–1.83)	0.238	-	-
Hispanic	0.98 (0.59–1.61)	0.925	-	-
Asian	0.83 (0.44–1.57)	0.558	-	-
AIAN	-	-	-	-
NHOPI	-	-	-	-
BMI	0.99 (0.97–1.01)	0.155	-	-
ASA (1–2)	0.49 (0.32–0.74)	0.001 *	REF	REF
ASA (3–4)	2.05 (1.35–3.10)	0.001 *	1.76 (1.14–2.71)	0.010 *
Electrolyte abnormality	1.16 (0.86–1.56)	0.344	0.99 (0.72–1.36)	0.943
Emergency case	1.75 (1.32–2.33)	<0.001 *	1.64 (1.22–2.20)	0.001 *
Smoking	1.25 (0.94–1.66)	0.127	1.22 (0.90–1.64)	0.203
Dyspnea	2.09 (1.39–3.13)	<0.001 *	1.76 (1.15–2.70)	0.009 *
Dependent functional status	1.48 (1.03–2.12)	0.033 *	1.22 (0.83–1.78)	0.310
Bleeding disorder	1.81 (1.17–2.81)	0.008 *	1.32 (0.82–2.12)	0.249
Anemic	1.59 (1.23–2.05)	<0.001 *	1.50 (1.13–1.98)	0.005 *
RBC transfusion day of surgery	1.23 (0.94–1.60)	0.126	1.13 (0.86–1.49)	0.384

OR: odds ratio; CI: 95% confidence interval; aOR: adjusted odds ratio; NHW: non-Hispanic white; NHB: non-Hispanic black; AIAN: American Indian or Alaska native; NHOPI: native Hawaiian or Pacific islander; BMI: body mass index; ASA: American Society of Anesthesiologists classification; RBC: red blood cell. * statistically significant.

**Table 5:** Impact of variables on non-routine discharge. Variables were included in univariate analysis if significant in Table 1, Table 2, Table 3 and Table 4 and N > 9. Variables were included in the multivariate model if *p* < 0.2 for univariate analysis.

Variable	Univariate		Multivariate			
	OR (CI)	*p*-Value	aOR (CI) Unsaturated	*p*-Value	aOR (CI) Saturated	*p*-Value
Malnourished	1.97 (1.58–2.47)	<0.001 *	1.78 (1.35–2.34)	<0.001 *	1.76 (1.34–2.32)	<0.001 *
Age	1.04 (1.03–1.05)	<0.001 *	1.04 (1.03–1.05)	<0.001 *	1.04 (1.03–1.05)	<0.001 *
Female	0.90 (0.74–1.10)	0.300	0.98 (0.76–1.26)	0.883	0.98 (0.76–1.26)	0.860
**Race**						
NHW	0.77 (0.60–0.99)	0.039 *	REF	REF	REF	REF
NHB	1.58 (1.14–2.18)	0.006 *	1.77 (1.23–2.54)	0.002 *	1.76 (1.22–2.53)	0.002 *
Hispanic	1.26 (0.84–1.90)	0.269	1.38 (0.87–2.19)	0.168	1.38 (0.87–2.19)	0.166
Asian	0.70 (0.42–1.17)	0.172	0.70 (0.39–1.26)	0.236	0.71 (0.39–1.28)	0.248
AIAN	0.61 (0.05–6.70)	0.683	0.66 (0.06–7.44)	0.739	0.68 (0.06–7.60)	0.752
NHOPI	1.21 (0.17–8.65)	0.846	0.72 (0.06–9.24)	0.804	0.74 (0.06–9.40)	0.815
BMI	1.00 (0.99–1.02)	0.878	-	-	-	-
ASA (1–2)	0.46 (0.34–0.63)	<0.001 *	REF	REF	REF	REF
ASA (3–4)	2.16 (1.58–2.96)	<0.001 *	1.13 (0.78–1.64)	0.530	1.12 (0.77–1.63)	0.547
Electrolyte abnormality	1.45 (1.13–1.87)	0.004 *	1.29 (0.94–1.76)	0.112	1.29 (0.94–1.76)	0.109
Emergency case	3.21 (2.45–4.20)	<0.001 *	4.02 (2.80–5.76)	<0.001 *	3.98 (2.77–5.71)	<0.001 *
Smoking	1.14 (0.89–1.45)	0.310	-	-	-	-
Dyspnea	1.05 (0.72–1.55)	0.793	-	-	-	-
Dependent functional status	1.96 (1.41–2.72)	<0.001 *	2.25 (1.47–3.44)	<0.001 *	2.25 (1.47–3.46)	<0.001 *
Bleeding disorder	2.43 (1.59–3.73)	<0.001 *	2.27 (1.35–3.83)	0.002 *	2.27 (1.34–3.82)	0.002 *
Anemic	1.53 (1.24–1.88)	<0.001 *	1.10 (0.84–1.43)	0.492	1.09 (0.84–1.43)	0.508
RBC transfusion day of surgery	1.35 (1.08–1.70)	0.009 *	1.48 (1.12–1.96)	0.006 *	1.45 (1.09–1.91)	0.010 *
Wound dehiscence	0.87 (0.24–3.08)	0.825	-	-	-	-
DVT	1.99 (1.23–3.22)	0.005 *	-	-	1.60 (0.93–2.75)	0.090
Systemic sepsis	2.28 (1.08–4.83)	0.031 *	-	-	1.18 (0.46–3.03)	0.735
Unplanned readmission	1.16 (0.88–1.53)	0.290	-	-	-	-

OR: odds ratio; CI: 95% confidence interval; aOR: adjusted odds ratio; NHW: non-Hispanic white; NHB: non-Hispanic black; AIAN: American Indian or Alaska native; NHOPI: native Hawaiian or Pacific islander; BMI: body mass index; ASA: American Society of Anesthesiologists classification; RBC: red blood cell; DVT: deep vein thrombosis. * statistically significant.

**Table 6:** Impact of variables on unplanned readmission. Variables were included in univariate analysis if significant in Table 1, Table 2, Table 3 and Table 4 and N > 9. Variables were included in the multivariate model if *p* < 0.2 for univariate analysis.

Variable	Univariate		Multivariate			
	OR (CI)	*p*-Value	aOR (CI) Unsaturated	*p*-Value	aOR (CI) Saturated	*p*-Value
Malnourished	1.79 (1.34–2.40)	<0.001 *	1.51 (1.11–2.05)	0.009 *	1.42 (1.04–1.95)	0.028 *
Age	0.99 (0.98–1.00)	0.119	0.99 (0.98–1.00)	0.024 *	0.99 (0.98–1.00)	0.033 *
Female	0.85 (0.64–1.12)	0.251	1.00 (0.74–1.35)	0.988	1.02 (0.75–1.39)	0.886
**Race**						
NHW	1.14 (0.81–1.61)	0.458	-	-	-	-
NHB	1.00 (0.65–1.56)	0.987	-	-	-	-
Hispanic	0.77 (0.42–1.41)	0.406	-	-	-	-
Asian	0.84 (0.41–1.73)	0.638	-	-	-	-
AIAN	2.65 (0.24–29.31)	0.428	-	-	-	-
NHOPI	-	-	-	-	-	-
BMI	0.99 (0.97–1.01)	0.246	-	-	-	-
ASA (1–2)	0.55 (0.34–0.89)	0.015 *	REF	REF	REF	REF
ASA (3–4)	1.82 (1.12–2.94)	0.015 *	1.63 (0.99–2.67)	0.053	1.59 (0.97–2.62)	0.067
Electrolyte abnormality	1.22 (0.86–1.72)	0.263	-	-	1.11 (0.77–1.60)	0.581
Emergency case	0.84 (0.58–1.23)	0.374	-	-	-	-
Smoking	0.91 (0.64–1.29)	0.584	-	-	-	-
Dyspnea	1.45 (0.89–2.37)	0.133	1.27 (0.76–2.10)	0.359	1.17 (0.69–1.97)	0.557
Dependent functional status	0.94 (0.59–1.49)	0.780	-	-	-	-
Bleeding disorder	2.12 (1.31–3.41)	0.002 *	1.89 (1.16–3.10)	0.011 *	1.90 (1.15–3.14)	0.012 *
Anemic	1.97 (1.44–2.70)	<0.001 *	1.78 (1.27–2.49)	0.001 *	1.74 (1.24–2.45)	0.001 *
RBC transfusion day of surgery	1.02 (0.74–1.40)	0.907	0.83 (0.60–1.16)	0.274	0.75 (0.54–1.05)	0.099
Wound dehiscence	4.95 (1.50–16.34)	0.009 *	-	-	3.31 (0.86–12.82)	0.083
DVT	3.98 (2.43–6.53)	<0.001 *	-	-	3.80 (2.28–6.34)	<0.001 *
Systemic sepsis	3.32 (1.57–7.02)	0.002 *	-	-	2.47 (1.13–5.42)	0.024 *
Non-routine discharge	1.16 (0.88–1.53)	0.290	-	-	-	-

OR: odds ratio; CI: 95% confidence interval; aOR: adjusted odds ratio; NHW: non-Hispanic white; NHB: non-Hispanic black; AIAN: American Indian or Alaska native; NHOPI: native Hawaiian or Pacific islander; BMI: body mass index; ASA: American Society of Anesthesiologists classification; RBC: red blood cell; DVT: deep vein thrombosis. * statistically significant.

## Data Availability

Data are contained within the article.
